# The human immunosenescence phenotype: does it exist?

**DOI:** 10.1007/s00281-020-00810-3

**Published:** 2020-08-05

**Authors:** Graham Pawelec

**Affiliations:** 1 Department of Immunology, University of Tübingen, Tübingen, Germany; 2 Health Sciences North Research Institute, Sudbury, ON Canada

## Abstract

“Immunosenescence” has been invoked as the root cause of increased incidence and severity of infectious disease in older adults and their poorer response to vaccination, and is implicated in increased solid cancers and increased autoimmunity with age. But how to define it in the individual and to show that immunosenescence is responsible for these adverse health outcomes? How can we monitor interventions aimed at restoring appropriate immune function to overcome these perceived immune deficits? Hence, the many efforts over the years aimed at establishing biomarkers of immunosenescence which to be useful must exhibit robust correlations with the chosen clinical outcome. Developments in “omics” technologies acquiring unprecedently detailed data on personal trajectories of immunosenescence and taking into account the under-appreciated importance of gender, ethnicity geography, socioeconomic, and multiple other differences will be of pivotal importance to identify biomarkers that are clinically useful at the level of the individual. This contribution addresses the question of whether or not we are currently in possession of any such useful biomarkers.

## Introduction

In a seminal paper published in 1962, Roy Walford proposed that mutations accumulating with age resulted in immune responses against newly emerging self-antigens, causing autoimmunity [1]. He developed this idea into an explanation of immune-mediated tissue damage generally as a cause of organismal aging in his famous book “The immunologic theory of aging” published in 1969 and in several associated publications [2]. These ruminations did a great service in drawing attention to altered immune responses with age, especially in the context of autoimmunity and transplantation immunity. However, they did not actually discuss alterations to immunity itself, and it was not until 1978 that the term “immunosenescence” first appeared in PubMed [3], although its use may have preceded this date [4], still in the context of discussions of autoimmunity, immunosuppression, and transplantation. The term very soon came to mean exclusively altered immunity in the aged, and many studies began to compare immunological parameters between younger and older individuals in cross-sectional investigations [5], and efforts to “rejuvenate” immunity soon followed [6].

## Caveats and constraints

Most human data to do with age and immunity are on circulating immune cells from peripheral blood, and many concern phenotypes expressed as percentages of subpopulations rather than absolute numbers per unit of blood, leading to the argument that a difference or a change in percentages can occur without any change in absolute numbers and therefore percentages are meaningless. This argument misses the fact that both absolute numbers and percentages are both only biomarkers and are therefore equally meaningful or meaningless unless closely correlated with a robust measurable outcome; they can only ever hint at mechanisms. Additionally, differences in absolute numbers of peripheral blood cell phenotypes do not imply differences in absolute cell availability throughout the organism because most immune cells are not circulating. Hence, comparisons of younger and older individuals are meaningful as generators of biomarkers for a particular measured state, say, for the sake of example, higher percentages of peripheral regulatory T cells (Tregs) in older adults might correlate with decreased delayed-type hypersensitivity (DTH) in cross-sectional studies and provide an indication of phenotype-outcome correlations. In this example, this likelihood could then be made stronger by demonstrating that percentages of Tregs increase over time in the same individuals as they age, and that DTH concordantly decreases, in longitudinal studies. In fact, however, there are vanishingly small numbers of even such imperfect such studies so far, and most studies show differences between young and old often interpreted as contributing to immunosenescence and deleterious outcomes but without demonstrating that this is in fact the case. Longitudinal studies are an improvement over cross-sectional studies but still only provide biomarker data. In humans, the only way to prove that changes to a proposed “immunosenescence phenotype” (IMP) are mechanistically linked to the measured outcome is to perform controlled intervention trials assessing the impact of the intervention on the IMP and at the same time on the clinical measure. In the present contribution, an attempt is made to review published human data on potential IMPs and to explore those studies that may be informative according to the above strict criteria.

## Early studies on human immunosenescence

As pointed out above, immune parameters acting as biomarkers for some measurable detrimental outcome of immunosenescence based on longitudinal studies of the same individuals over time are superior to cross-sectional studies for establishing clinical relevance and are of course essential for monitoring the impact of interventions. Amongst many challenges to such studies in humans, there is also a certain challenge in selecting the outcome measure to be considered as the endpoint. Here, mortality is the most unequivocal outcome (albeit with a whole raft of its own problems related to cause of death). Already in 1974, Roberts-Thompson et al. had reported an association between weaker DTH reactions and mortality at 2-year follow-up in people over 80 years of age at baseline [7]. However, this study remained an isolated case more or less until a decade later, when some early human work leveraged ongoing programs by retrospectively analyzing simple immunological parameters such as absolute peripheral blood lymphocyte counts and correlating them with mortality in longitudinal studies like the pioneering Baltimore Longitudinal Study of Aging (BLSA) [8]. A very recent publication reported on a study started in 1979, recording responses to the mitogen PHA in 246 subjects aged at least 65 at baseline, concluding that differences in baseline T cell responses contributed to a 4-year survival difference at a mean follow-up of 36.5 years [9]. A Japanese study followed subjects aged 69–72 years at baseline over 7 years and identified a significant association between decreases in polymorphonuclear leukocyte chemotaxis and mortality [10]. Probably the longest follow-up studies on a human population analyzed Hiroshima atom bomb survivors, admittedly not exactly a normal population, but where some evidence of earlier changes to T cell immunity was forthcoming, for example, in 1991 after 45 years [11]. Consistent with findings from cross-sectional studies concerning reduced T cell proliferative responses to mitogens [12] (which even at that time were challenged by other findings that attributed decreased responses, not to chronological age alone but also state of health [13]), results from early longitudinal studies indicated that T cell responses were prognostic for all-cause mortality. The pioneering Swedish OCTO study of free-living very old people selected for excellent health included some immune components, examining mitogen responses and CD3+ T cell counts, and finding that decreased absolute counts of the latter were progressively seen with increasing age [14]. Shortly thereafter, the Swedish study published an analysis of extended immune parameters which correlated with mortality on a 2-year follow-up of 102 individuals aged 86–92 years at baseline. This important study was the first to find that a cluster of parameters but not any single value by itself predicted worse survival. These factors included poor T cell proliferative responses to mitogens, high CD8+ T cell percentages, and low CD4+ T cell and CD19+ B cell percentages. Furthermore, these investigators resampled and re-examined subjects after a subsequent 2-year period, itself rarely accomplished even in longitudinal studies. This revealed that certain individuals acquired the constellation of markers associated with increased mortality over the 2-year period, reflected in progressively decreasing CD4:8 ratios due to excessive accumulations of CD8+ T cells [15]. The last follow-up of the OCTO study 8 years after starting was in the meantime able to include some slightly more sophisticated surface marker phenotyping, reporting that non-survivors exhibited decreased numbers of CD4+ T cells and increased CD8+ T cells, particularly those lacking the positive costimulatory receptor CD28 but expressing CD57, usually found on natural killer cells. Strikingly, these alterations were greatly amplified in subjects infected with the persistent human herpesvirus 5 (cytomegalovirus, CMV) [16]. This cluster of parameters associated with incipient mortality in very old subjects came to be known as the “immune risk profile” (IRP) [17]. It was shown in the parallel NONA study of a representative population from the same location but unlike OCTO, not selected for excellent health, the IRP remained predictive of all-cause mortality even in mixed populations including frail subjects [18, 19]. Finally, studies on the same Swedish populations indicated that several additional factors, especially those reflecting an increased systemic inflammatory condition linked with cognitive impairment, were at least additive with the IRP and possibly even synergistic, in terms of the strength of their association with mortality [20]. Thus, these early studies seemed to establish that certain constellations of immune-related parameters, here collectively termed the “immunosenescence phenotype” or IMP (i.e., the IRP plus non-IRP factors), were associated with earlier all-cause mortality in the very elderly. However, many questions were left open. These included the crucial question of whether the same risk factors applied to younger populations and not only to highly selected oldest old adults as in the Swedish studies, whether the same IRPs and IMPs applied to people in other countries under different circumstances, whether they applied similarly to all birth cohorts, and many others, especially whether an improved extended IMP could be determined that would be informative at the level of the individual. In the following, an attempt will be made to answer some of these questions. The focus will be only on those studies assessing the relevance of these biomarkers for the unequivocal outcome of mortality, without including the much more numerous studies on their impact on frailty, which is outside the scope of this overview and would require a separate special issue.

## IMPs and IRPs

As discussed above, IMPs include potentially immune-related parameters additional to those primarily cellular components of the original IRP. These other factors may not be exclusively immune-related (they can be inflammatory mediators that may or may not have an immune origin, e.g., they may be derived from senescent cells as part of the senescence-associated secretory phenotype or SASP [21]). These factors will be included here in the review of IMPs. To reiterate, to be included in this discussion, human IMPs are defined as any potentially immune-related biomarkers that have convincingly been shown to correlate with age-associated mortality. First, I will focus on our own efforts primarily in collaboration with Prof. A. Wikby et al. in Jönköping, Sweden (OCTO and NONA longitudinal studies), Prof. R. Westendorp et al. in Leiden, The Netherlands (Leiden 85-Plus and Leiden Longevity Studies), and Prof. C. Mathei et al. in Leuven, Belgium (BELFRAIL study).

### Swedish OCTO/NONA and considerations deriving from these studies

In addition to the simple IRP already mentioned, further work revealed that the accumulations of late-stage differentiated CD8+ T cells responsible for the inverted CD4:8 ratio were to a large extent specific for CMV, but with evidence of dysfunctionality in that a much lower fraction of such cells secreted interferon-γ when specifically stimulated [22]. However, such was the degree of enrichment for these cells that their absolute numbers were higher in the elderly than in younger subjects. We therefore suggested that their accumulation was a compensatory measure to maintain essential immunosurveillance against latent CMV, which may be a common theme in aging. This notion was supported by the finding that progressive loss at advanced age of the predominantly CMV-specific clonal expansions of CD8+ T cells was associated with incipient mortality [23]. As a simple surrogate for the IRP, merely the presence of an inverted CD4:8 ratio as been employed in several studies by other investigators, this is not definitive and may help explain why some reports are consistent with its predictive value [24, 25] but others not [26]. As it is CMV that predominantly drives the inverted CD4:8 ratio and potentially other factors of the IMP [27], associations with mortality may be reflecting differential susceptibility of different populations to these effects [28]. Herein lies another important general lesson: IMPs may be highly specific for particular populations and even different birth cohorts within the same population, and certainly for women and men, such that making generalizations is hazardous [29]. Hence, our and others’ efforts to improve and validate the IRP and IMPs in general may be fated to have relevance restricted mostly to the specific population studied, and the value of these efforts to establish consensus predictive biomarkers may be questionable. It is noteworthy that one parameter almost universally agreed upon in the field is that older adults possess far fewer naïve T cells in the blood (especially CD8+ cells) both in terms of absolute numbers and percentages. However, few if any studies have assigned actual clinical relevance to these findings, and low levels of naïve CD8+ T cells were not included in the cluster of parameters defining the IRP in OCTO/NONA. It therefore remains only an assumption that possessing fewer naïve cells (in the blood) predicts a poor prognosis, for example, on a challenge with a novel infectious agent. Of the few studies in humans, for example on vaccination of older people for yellow fever, poorer antibody responses did correlate with a dearth of CD4+ naive T cells and with dysfunction of antigen-presenting cells (APCs) [30], consistent with this hypothesis.

### Leiden 85-plus and Leiden familial longevity studies

The ground-breaking Leiden familial longevity studies (LLS) indicated that there is a 30% reduction in the standardized mortality rate of the second-generation offspring of exceptionally long-lived parents and grandparents, which suggests that their lifespans will be extended relative to the other 99.5% of the Dutch population [31]. This is indeed the case for nonagenarians in the LLS relative to sporadic nonagenarians [32]. It was interesting to note that although the probability of becoming infected with CMV was slightly lower in the offspring [33], the impact of CMV on driving the accumulation of late-stage CD8+ T cells was reduced or absent in these subjects, although there was no effect on the proportions of naïve T cells [28]. This may contribute to their exceptional longevity, because these are the cells that assigned at the least the Swedish OCTO/NONA participants to the IRP group. Again illustrating the relative possible unimportance of naïve T cells, in a small study of participants in the Leiden 85-Plus cohort, with very limited availability of cryopreserved cells from earlier in the study, we were able to show that at a very advance age (89 at baseline), 7-year survival associated with higher levels of CMV-reactive memory cells but not naïve T cells [34]. Moreover, assays showed that specific functionality of these cells in terms of their production of pro-inflammatory factors but lack of production of anti-inflammatory factors on in vitro stimulation with CMV antigens was the deciding factor in the association with remaining survival [34]. We interpreted these results as suggesting that at the very late stage of life (in Leiden, The Netherlands, at least), continued survival was facilitated by the presence of pre-inflammatory anti-CMV responses that perhaps contributed to the essential ability to prevent latent CMV from reactivating. An enhanced pro-inflammatory state is considered detrimental in many studies, but our finding that survival in this 85-plus cohort was positively associated with higher levels of Tregs that could dampen systemic inflammatory responses may represent a compensatory mechanism protective against this necessary anti-CMV response [35]. All of these considerations of course remain speculative interpretations of biomarker data, as pointed out at the beginning of this essay.

### BELFRAIL

The above-mentioned results from the Swedish and Dutch studies could be interpreted to suggest that multifactorial IMPs that correlate with survival at least in very elderly northern Europeans are more to do with the necessity for constant immunosurveillance against CMV (but not other common herpesviruses [36]) than anything else. Due inter alia to the extremely limited availability of biobanked viably cryopreserved PBMC in the above studies, we sought additional studies in which to further test and extend the IRP and determine whether more informative IMPs could be constructed. To this end, we initiated a collaboration with BELFRAIL, which was a prospective population-based cohort study that examined 567 subjects selected only to exclude those with severe dementia or acute disease who were 80 years of age at baseline [37]. This study examined the effects of CMV infection in early analyses of physical and cognitive function and concluded that it was not associated with functional impairment in either domain, although high titers of anti-CMV IgG tended towards such an association [38]. Consistent with this, CMV infection as a single factor was not related to mortality on subsequent follow-up, although individuals with the highest antibody titers were at higher risk for all-cause mortality [39]. Subjects with the highest levels of the commonly used marker of systemic inflammation, interleukin 6 (IL 6) were more likely to exhibit physical and cognitive impairment [40]. Due to the availability of cryopreserved PBMC from nearly 300 of the BELFRAIL participants at baseline (a rare resource at that time), it was possible to ask whether the CD4:8 ratio was informative in this cohort as in the Swedish OCTO/NONA. The results were striking. They did show that the CD4:8 ratio was informative, but in contrast to OCTO/NONA, an inverted ratio correlated with better survival, whereas a ratio > 5 was associated with poorer survival. Even more striking was that a positive contribution of CMV seropositivity was seen, and the 3-year survival of women with an inverted CD4:8 ratio who were CMV-infected was significantly better than of those with either marker alone and especially of those without either the inverted ratio or CMV infection [41]. Quite remarkable was the finding that in men, there was absolutely no difference in 3-year survival according to any of these parameters. It is well-accepted that many immune parameters are different between men and women, but to the best of my knowledge, the BELFRAIL findings provide the most dramatic illustration of such clinically relevant sex effects. But how do these diverse OCTO/NONA-vs-BELFRAIL findings compare with studies in other cohorts?

## Longitudinal studies including potential immune biomarkers in other cohorts

There have been, and are, several longitudinal studies that include or included certain potential IMP components. Those biomarkers most commonly included pertain to markers of inflammation, particularly CRP and IL 6. These are the factors primarily measured to assess “inflammaging” [42] which were informative for longevity for example in the Rancho Bernado study [43]. Interestingly, this study found that higher levels of IL 6 or CRP were associated with shorter remaining survival and shorter lifespan in men, but not generally in women, another example of a stark difference between the sexes [43]. In the PolSenior study, both CRP and IL 6 levels remained predictors of 5-year survival after correction for sex [44]. Many factors influence levels of IL 6, including genetic polymorphism, which have been shown for example in longitudinal studies of Danish populations to associate with longevity [45]. Nonetheless, associations with CRP appear universal across different populations, with similar results reported in Asians as well as the more commonly investigated Caucasians [46]. However, examining single factors for association with mortality rarely yields strong predictive ability and multiple factors need to be taken into account, a recurring theme in all these studies. For example, in the In-Chianti study, a composite of 19 inflammatory markers was most informative, although the level of IL 6 by itself was indeed still a useful predictor of mortality [47]. More recently, several studies have begun to sample a broader set of parameters including cellular immune values as well as a range of genetic, physical, medical, psychosocial, and cognitive data in an attempt to gain a comprehensive view of factors associated with healthspan and lifespan. Many of these studies, such as the Berlin BASE II study [48], currently have baseline data but not yet long-term follow-up. However, some studies have reached the stage of maturity where data can be scrutinized in the search for IRPs, IMPs, and general holistic predictors of healthspan and lifespan. Some of these are discussed below, without any claim to comprehensive coverage of the entire field.

### The Newcastle 85-plus study

The N85+ (Newcastle 85-plus) was designed as a longitudinal study of 85-year-olds from the northern UK recruited at baseline from the 1921 birth cohort and re-examined after 1.5 and 3 years. A broad range of outcome measures was planned, including questionnaires on socio-economic status, physical and psychological health, diet, and lifestyle, and including extensive blood tests for routine hematology and biochemistry, lipid profiles, thyroid function, inflammatory markers (CRP, TNF, IL 6), cortisol, biomarkers of DNA repair capacity, telomere length, and lymphocyte subpopulation distributions. DNA, RNA, and plasma were biobanked. Mortality has been recorded since the study started in 2006 [49]; while many papers have been published in the meantime, relatively few have dealt with immunological markers. Where these have correlated parameters relevant to IMPs and IRPs with mortality, diverse outcomes have been reported. An early analysis 1.5 years from baseline suggested that mortality was not associated with “several proposed biomarkers of ageing, notably inflammation and immune risk markers and telomere length.” [50] However, this follow-up period may have been too short, and the authors concluded that “As future data accrues on health outcomes within the cohort, it will become possible also to evaluate the predictive value of these and others of the candidate biomarkers.” [50] Indeed, a 7-year follow-up revealed that clusters of biomarkers did correlate with survival [51]. Many of the immunological parameters included in this “Frailty Index” (FI) were remarkably similar to those identified as associated with survival in the Swedish OCTO/NONA studies (inverted CD4:8 ratio due to an accumulation of CD8+ TEMRA cells, higher CRP, more neutrophils, lower albumin) and included some parameters not tested for in the former (e.g., low TGF-ß) [51]. Interestingly, however, unlike in OCTO/NONA, CMV seropositivity was not associated with the overall FI, and was not analyzed together only with the immunological parameters. CMV status was not the only “discrepancy” because IL 6 was not included either [51]. Hence, one could conclude that the N85+ results are mostly consistent with the IRP but divergent regarding IMPs and the exact relationship between frailty and mortality. These findings emphasize the confounding effects of CMV, which remain controversial. Later analyses of the N85+ cohort did reveal specific effects of CMV on mortality due to coronary heart disease (CHD) which were associated with accumulations of CD8+ TEMRA cells and lower CD4:8 ratios [52]. Very recently, a new 7-year follow-up suggested a possible reason for the divergence of CMV from predictive IMPs in some studies of some cohorts [53]. In that study, it was found that CMV-independent accumulation of CD8+ TEMRA cells of the earlier differentiation phenotype (CD27-CD28+ rather than the double-negative “senescent” cells which were driven by CMV) appeared to be positively associated with lower mortality [53]. Memory CD8+ T cells with this phenotype would be expected to be less likely to exhibit a SASP and to be more likely to retain effector function against their specific targets, not CMV in this case. These new data are intriguing and further results are eagerly awaited.

### CARRERITAS

In this Spanish cohort over 65 years of age at baseline (mean age 80), a 2-year follow-up revealed that an inverted CD4:8 ratio as a surrogate of the IRP was associated with excess mortality [54]. As in the Swedish OCTO/NONA studies, high neutrophils, CRP, and IL 6 were also associated with mortality. This study included an assessment of recent thymic emigrants by quantifying T cell receptor excision circles (TRECs) and found a strong correlation between higher TRECS and better survival, consistent with the importance of higher levels of naïve T cells in this cohort (not seen using surface markers in the Swedish studies). Multivariate analysis of this dataset revealed that only CRP and TREC levels were independently associated with survival [54]. A more detailed analysis of the late-stage differentiated CD8+ TEMRA cells showed that the accumulation of dysfunctional CMV pp65-reactive cells was also associated with 2-year mortality [55], consistent with data from OCTO/NONA and Leiden 85+ studies [34, 56, 57].

### Stanford clinical and translational research unit cohort

A recent seminal study has applied “multi-omics” approaches in a cohort of 135 younger and older adults repetitively sampled over 9 years as part of an influenza surveillance project. This method was able to capture change over time and associate immune parameters with overall survival, which was remarkably independent of age, sex, or cardiovascular disease. Amongst other things, this revealed the fascinating finding that rates of change of immune parameters in different individuals were independent of age but were mostly fixed from the beginning. This approach resulted in the definition of a measure of changing trajectories of immune markers with age, which were mostly those candidates identified in earlier studies, as illustrated above. Moreover, this immune signature, denoted as “IMM-AGE,” was validated in a well-characterized 3rd party cohort, the Framingham Heart Study, and shown to predict mortality more accurately than other established biomarkers [58].

## Conclusions

The results briefly reviewed here provide a stark illustration of the context dependency of any associations of immune risk profiles with survival in late life. Any potential explanations of these sometimes dramatic differences remain entirely speculative at this stage, but is highly likely that many differences are related to the individual’s history of immune reactivities to past exposures over the lifespan (that has been termed their “immunobiography” [59]). The only potentially immune-related biomarker parameter that appears universally associated with mortality across populations in multiple studies is the slightly higher IL 6/CRP level in serum—and this may not even have an immune cell origin, but could be derived from senescent non-immune cells [60]. Lower numbers and proportions of naïve CD8+ T cells in the blood also seem to be universally observed, but have not been shown to be robust markers of mortality in humans. The accumulation of CD8+ CD28− T cells, as discussed above, and commonly reported in many studies, may still be the closest we can get to a simple robust marker, but with many variables and caveats as discussed above, and predominantly driven by latent CMV infection. The implication is that if they are ever to be informative at the level of the individual, IMPs will need to include a large number of factors influential throughout life, but each of which makes only a small contribution by itself. Even if a personalized IMP informative for individuals within a population could be determined, it would still be the case that such signatures might not be relevant for younger members of the same populations, given the changed circumstances experienced by different birth cohorts. Hence, environmental and developmental variables could impose effects at the epigenetic level (theoretically amenable to analysis), genetic differences, and psychosocial effects as well as pathogen exposures could all contribute to rendering this undertaking intractable. The answer to the question assigned for the title of this article “The Human Immunosenescence Phenotype: does it exist?” must then be no, it does not—but many do, which must be matched to the circumstances and the context, and might no longer be relevant for future birth cohorts. The IMM-AGE metric using “omics” approaches mentioned above to collate multiple relevant immune parameters is very promising in accomplishing the former aim and answering that question once other non-immunological components influencing the immune biomarkers have also been integrated into it. Such fascinating studies already exist, but usually without much in the way of immune markers [61]. Marrying all these data together promises to provide a more accurate answer to the question of whether the IMP is the overriding factor or dependent on other health states, particularly the heterogeneous condition referred to as “frailty.” Nonetheless, it seems to me that the question of whether any such IMPs that are informative for current birth cohorts will necessarily have identical predictive value for other birth cohorts may be intractable. However, a major crucial clinical application of individualized IMPs will be as biomarkers for predicting responses to infections and interventions such as vaccinations, for example, the current urgent requirement for assessing immune status in older adults in the context of the emergence of the novel coronavirus SARS-CoV-2 [62, 63].
